# Mitochondrial outer membrane permeabilization at the single molecule level

**DOI:** 10.1007/s00018-021-03771-4

**Published:** 2021-02-12

**Authors:** Shashank Dadsena, Andreas Jenner, Ana J. García-Sáez

**Affiliations:** 1 Institute for Genetics, CECAD Research Center, University of Cologne, Cologne, Germany; 2 Interfaculty Institute of Biochemistry, University of Tübingen, Tübingen, Germany

**Keywords:** Apoptosis, Bcl-2 proteins, Mitochondrial outer membrane permeabilization (MOMP), Single-molecule techniques

## Abstract

Apoptotic cell death is essential for development, immune function or tissue homeostasis, and its mis-regulation is linked to various diseases. Mitochondrial outer membrane permeabilization (MOMP) is a central event in the intrinsic apoptotic pathway and essential to control the execution of cell death. Here we review current concepts in regulation of MOMP focusing on the interaction network of the Bcl-2 family proteins as well as further regulatory elements influencing MOMP. As MOMP is a complex spatially and temporally controlled process, we point out the importance of single-molecule techniques to unveil processes which would be masked by ensemble measurements. We report key single-molecule studies applied to decipher the composition, assembly mechanism and structure of protein complexes involved in MOMP regulation.

## Current concepts in mitochondrial outer membrane permeabilization, a key process in apoptosis

Apoptosis is the most studied and best understood form of regulated cell death [30]. In mammalian cells, apoptosis is sub-classified into two major signaling pathways; the mitochondria mediated intrinsic pathway and the death receptor mediated extrinsic pathway. In the intrinsic pathway, diverse apoptotic stimuli convey death signals to mitochondria [16, 97] eventually resulting in the release of soluble proteins like cytochrome *c* and Second mitochondria-derived activator of caspase (Smac) from the mitochondrial intermembrane space (IMS) to the cytosol as a consequence of MOMP. MOMP controls the execution of the cell death program and it is considered the point of no return in the cellular commitment to death [24, 92, 103]. Recent studies have revealed that mitochondrial DNA is also released from mitochondria upon MOMP, which in the absence of caspase activity initiates inflammatory signaling via the cGAS/STING pathway, thereby linking MOMP to the regulation of the inflammatory outcome of apoptosis [65, 81].

MOMP is regulated by the proteins of the B-cell lymphoma 2 (Bcl-2) family and leads to the activation of caspases that execute cleavage of a target set of cellular substrates resulting in dismantling of the cell and finally cell death [47, 102]. There are around 20 members in the Bcl-2 family that form a complex interaction network not only in solution but also in cellular membranes, and whose balance determines the outcome of MOMP and apoptosis induction. Due to the complexity of this signaling hub, we still fail to fully understand how the Bcl-2 proteins determine cell fate.

The proteins of the Bcl-2 family are subdivided into three groups based on their apoptotic function and their domain organization [13, 64]. Anti-apoptotic (pro-survival) Bcl-2 proteins have four Bcl-2 homology (BH) domains (BH1-BH4), mostly integrated within the mitochondrial outer membrane (MOM) and include Bcl-2-related gene A1 (A1), Bcl-2, BCL-2-related gene, long isoform (Bcl-xL), Bcl-w, and myeloid cell leukemia 1 (Mcl-1) [13]. Proteins of this subfamily preserve MOM integrity by directly inhibiting the pro-apoptotic Bcl-2 family members, either on the mitochondrial membrane or in the cytosol. Pro-apoptotic Bcl-2 effector proteins also have four BH domains and include the Bcl-2-associated X protein (Bax), Bcl-2 antagonist/killer protein (Bak) and likely the Bcl‑2‑related ovarian killer protein (Bok). In mammalian cells, Bax and Bak are considered as the master effectors of MOMP. Upon apoptosis induction, Bak and Bax accumulate in mitochondria and homo- and/or hetero-oligomerize into proteolipidic pores within the MOM to promote its permeabilization [5, 6]. Activation of either Bax or Bak is essential for MOMP as cells lacking both proteins fail to undergo MOMP in response to many stresses [107]. Finally, pro-apoptotic BH3-only proteins constitute the by far largest subgroup in the Bcl-2 family. Well-known members of this subfamily include Bid, Bim, Puma, Bad and Noxa [50]. BH3-only proteins provide a crucial link between apoptotic stress stimuli and the mitochondrial apoptotic machinery. For instance, in response to DNA damage that cannot be repaired, cells accumulate the tumor suppressor protein p53, a transcription factor that stimulates expression of Puma and Noxa. These BH3-only proteins initiate apoptosis by inhibiting the pro-survival Bcl-2 proteins and some of them also by directly triggering the activation of Bax and Bak [10, 12, 22, 27].

Bak and Bax activation at the MOM is a crucial step in apoptosis initiation that under physiological conditions necessitates the contribution of BH3-only proteins to shift the balance between cell survival and death [11]. However, the function of BH3-only proteins in their interplay with other Bcl-2 family proteins is highly debated. Several models have been proposed to explain the interplay of Bcl-2 proteins in the regulation of apoptosis. The ‘direct activation model’ suggests that some BH3-only proteins (most prominently Bim and Bid) act as activators that can bind and directly activate Bax and Bak by inducing a conformational change that allows their oligomerization in the MOM, while the other BH3-only proteins (e.g., Bad) function as sensitizers and engage only anti-apoptotic proteins, thereby blocking their pro-survival role and reducing the threshold of apoptotic stimulation [49, 57]. An alternative ‘neutralization’ or ‘indirect activation model’ proposes that upon apoptosis induction, BH3-only proteins bind and neutralize antiapoptotic Bcl-2 proteins, which displaces and releases constitutively active Bax and Bak from their inhibitory complex with antiapoptotic Bcl-2 family proteins [8, 43, 108]. The ‘embedded together’ model, considers that the anti-apoptotic Bcl-2 proteins inhibit direct activators as well as the executors Bax and Bak while pointing out the importance of the membrane environment for protein–protein interactions [54, 55]. The ‘unified model’ built on the ‘embedded together’ model by proposing that the anti-apoptotic proteins sequester the activator BH3 proteins (MODE 1) as well as Bax and Bak (MODE 2). Inhibition of apoptosis through MODE 1 is proposed to be less efficient and, therefore, easier to overcome by sensitizer BH3 proteins. This model extends the role of Bcl-2 family proteins and the regulation of MOMP to mitochondria dynamics [59]. The more recent ‘interconnected hierarchical model’ [7] established a sequence of events for the execution of apoptosis. This suggests a bimodal mechanism for the step-wise inhibition of anti-apoptotic Bcl-2 family proteins and the activation of Bax/Bak by BH3-only proteins, in addition to the neutralization of BH3-only proteins and executors Bax/Bak by anti-apoptotic Bcl-2 proteins. Recently, genetic deletion of most Bcl-2 proteins and reintroduction of Bax alone reported the induction of MOMP in absence of any apoptosis stimulus. Although these cells still contained less well characterized Bcl-2 proteins (like Bok, BPR, Bcl-rambo, Bcl-G and BFK), this suggested that Bax activation does not entirely rely on endogenous BH3-only proteins [74], but may involve other unknown activation factors or, more intriguingly, the ability of Bax and Bak to auto-activate.

In healthy cells, Bax is a largely soluble monomeric protein that constitutively shuttles between the cytosol and the mitochondrial surface [26, 109]. Under these conditions, pro-survival Bcl-2 proteins like Bcl-xL antagonize Bax by promoting its retrotranslocation from mitochondria into the cytosol. Bak is also subjected to retrotranslocation. However, the predominant mitochondrial Bak and largely cytosolic Bax localizations result from different retrotranslocation rates caused by a difference in the hydrophobicity of their *C*-terminal trans-membrane segments [98, 99]. Apoptosis induction blocks retrotranslocation of Bax by a BH3-only protein-mediated inhibition of pro-survival Bcl-2 proteins, resulting in an accumulation of Bax in the MOM. In its inactive soluble form, Bax has a globular structure comprising nine α-helices arranged around a central hydrophobic core composed of amphipathic helices α 2– α 5 [34, 96]. Induced, ‘direct activator’ BH3-only proteins (by upregulation or activation via post-translational regulation) interact with the mitochondrial pool of Bax, thereby promoting a series of conformational changes that releases its *C*-terminal trans-membrane segment from a hydrophobic groove [37, 109]. This enables Bax to insert into the MOM and to transiently expose its BH3-domain, a crucial step in promoting Bax oligomerization [35, 60]. Activated Bax and Bak form homodimers and heterodimers, which causes the release of BH3-only proteins and drives further dimer-by-dimer oligomerization [17, 23, 112]. As discussed above, the resulting Bak and Bax oligomers are believed to directly mediate MOMP. Recently, it has been revealed that Bax forms assemblies in the shape of arcs and rings on the surface of apoptotic mitochondria, whereas similar arc and ring-assemblies of Bax were found to penetrate model bilayers by atomic force microscopy [38, 85].

**Fig. 1:**
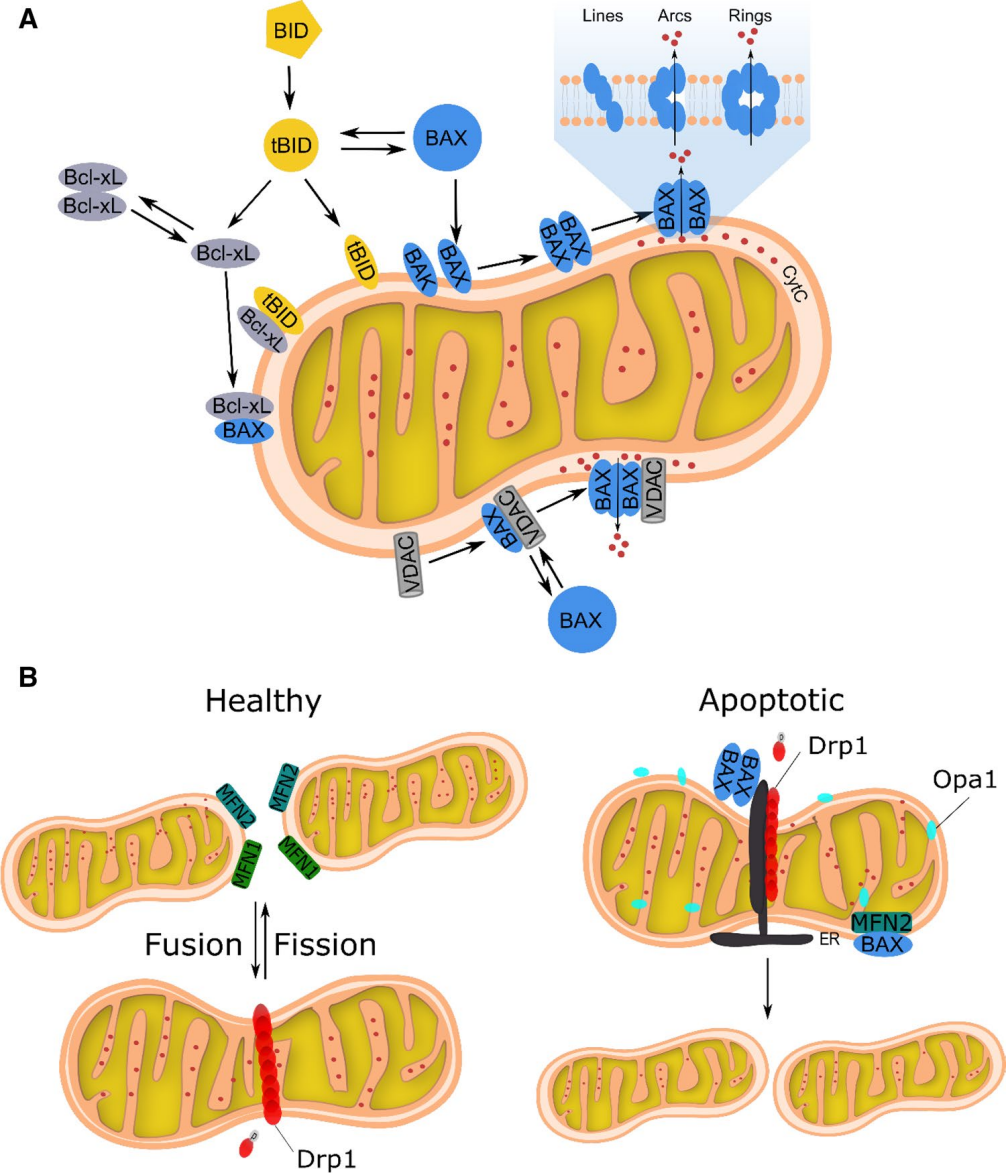
Schematic representation of regulatory processes mediating MOMP and mitochondrial dynamics under healthy or apoptotic conditions. **a** Activation model of Bax/Bak-mediated MOMP. Illustrated is the dynamic interplay of pro- and anti-apoptotic Bcl-2 proteins in controlling permeabilization of mitochondrial outer membrane (MOM). Due to a lower rate of retrotranslocation BAK is mostly MOM-bound. Besides Bcl-2 family proteins, VDACs also regulate Bax and Bak activity, likely with different outcomes. In the case of Bax, they serve as platform for its assembly on the surface of mitochondria and formation of Bax pores. **b** Mitochondrial dynamics imbalance during apoptosis. (Left; Healthy mitochondria) Mitochondrial fusion and fission proteins and their roles in changing mitochondrial morphology. (Right; mitochondria in apoptotic cells) Activation of Bax/Bak during apoptosis leads to an imbalance in mitochondrial dynamics towards fission and *cristae* remodeling

The fact that Bak and Bax oligomers primarily assemble in the MOM and not in the membranes of other cellular organelles raises the question of how their mitochondrial targeting is accomplished. Current research work indicates that this process involves other proteins such as members of a specialized class of porin ion channels in the MOM, namely the voltage-dependent anion channels (VDACs) [53, 88]. Subsequent work revealed that insertion of Bak into the MOM is dependent on VDAC2 [71, 83]. Following a systematic analysis of chimeric constructs, VDAC2 was found to be critical for Bak recruitment [71]. Another study demonstrated that VDAC2 is also required for Bax recruitment, but only when Bak is absent [63]. While VDAC2 appears to inhibit Bak activation and mitochondrial apoptosis [9], VDAC2-mediated Bak recruitment is a precondition for mitochondrial apoptosis induced by BH3-only protein tBid [83]. To reconcile these apparently conflicting findings, it has been proposed that VDAC2 can both stimulate apoptosis by mediating Bax/Bak recruitment to mitochondria and suppress apoptosis by maintaining Bax/Bak in a protein complex that hinders their activity [70, 101]. In line with this idea, a recent study showed that VDAC2 has a dual role by conferring MOM-specific Bax recruitment and ensuring Bax inhibition by promoting its retrotranslocation into the cytosol [52]. Consequently, VDACs are active participants in mitochondrial apoptosis by serving as dynamic platforms for Bax/Bak (retro)translocation and/or as essential components of apoptotic MOM pores (Fig. 1a).

During apoptosis, mitochondria undergo dynamic alterations in their structure and function. Besides MOMP, apoptotic mitochondria lose the trans-membrane potential and undergo remodeling of the inner membrane *cristae* and enhanced lipid transfer between the mitochondrial inner membrane (MIM) and the MOM as well as with the endoplasmic reticulum (ER) [28, 36, 42, 45, 104]. Additionally, mitochondria undergo massive fragmentation in apoptosis, converting the interconnected mitochondrial network to vesicular, punctate mitochondria [64, 95]. In healthy conditions, the structure of the mitochondrial network is determined by a dynamic equilibrium of well-balanced, continuous fusion and fission events governed by dynamin-like proteins. In mammals, the fission of mitochondria is mediated by the Dynamin-related protein 1 (Drp1), a large mechanochemical GTPase which shuttles between the cytosol and the MOM. During apoptosis, the key executors of MOMP, Bax and Bak, accumulate at mitochondria and redistribute into discrete foci, the so-called apoptotic foci, which colocalize with Drp1 and Mitofusin-2 (Mfn2) at mitochondrial constriction sites [46]. Although not yet quantitatively analyzed, these foci do not seem to freely diffuse, but rather to locate at concrete sites regularly distributed along the mitochondrial network. It is tempting to speculate that apoptotic foci form at defined cellular structures of specific molecular architecture. At least in healthy conditions, these membrane constrictions predominantly occur, where ER tubules wrap around mitochondria to mark mitochondrial fission sites [29]. We still do not understand what is the molecular composition and structural organization of apoptotic foci. For example, local alterations in the membrane curvature and lipid composition at these sites might be favorable for Drp1 assembly and Bax/Bak-mediated pore formation [111]. Bax and Bak-mediated SUMOylation of Drp1 promotes its stable association at the MOM, potentially promoting mitochondrial fragmentation [76, 106]. In line with this, studies in cell-free systems revealed that Drp1 stabilizes non-lamellar membrane hemi-fusion intermediates with high negative curvature that were shown to trigger Bid-induced Bax oligomerization [69]. However, the link between Drp1-mediated fragmentation in apoptosis and pore formation by Bax and Bak remains controversial as loss of Drp1 alters apoptosis kinetics but does not prevent MOMP and successive cell death [44].

The fusion of mitochondria is regulated by other dynamin-like proteins; two mitofusin proteins Mfn1 and Mfn2 located in the MOM and Optic atrophy protein 1 (Opa1) located in the MIM [15, 86]. The crystal structures of a minimal Mfn1 suggests that Mfn1 dimerizes in a GTP-dependent manner, causing membrane clustering, thereby, functioning as a tether between fusing mitochondria [78]. Mfn2 is mainly known for tethering mitochondria to the ER and its oligomerization stimulates mitochondrial fusion [21, 89]. Inhibition of mitochondrial fusion during apoptosis has been linked to the activity of different kinases that phosphorylate both mitofusins [56, 77]. Phosphorylation of Mfn1 was shown to inhibit mitochondrial fusion by perturbing Mfn1 oligomerization and mitochondrial tethering. Moreover, its phosphorylation is implicated in promoting Bak oligomerization to favor MOMP via yet obscure mechanisms [77]. Another target for mitochondrial fragmentation during apoptosis is Opa1. The role of Opa1 in mitochondrial fusion is attributed to coordination of MOM and MIM fusion. The pro-apoptotic BH3-only protein Bnip3 was shown to interact with Opa1 and to inhibit Opa1-mediated mitochondrial fusion. The interaction of Bnip3 and Opa1 triggers the disruption of the Opa1 complex in a Bax- and/or Bak-dependent manner, ultimately leading to mitochondrial fragmentation and apoptosis [51]. Cytochrome *c* resides within mitochondrial *cristae*, which are connected to the peripheral portion of the IMS by narrow *cristae* junctions. The tightness of *cristae* junctions correlates with oligomerization of Opa1, which protects from apoptosis by preventing cytochrome *c* release. Remodeling of *cristae* occurs concomitant with the release of cytochrome *c* upon MOMP. The pro-apoptotic Bcl-2 family member proteins Bid and Bim have been shown to disrupt Opa1 oligomers, which allows the widening of *cristae* junctions and enhances cytochrome *c* release [28, 110] (Fig. 1b).

Although apoptosis research has primarily focused on the role of Bcl-2 proteins, a growing body of evidence also supports a critical role of lipids. It is widely accepted that Bax/Bak oligomers and membrane lipids are key component of apoptotic pore, which has a proteolipidic nature [5, 6]. Cardiolipin has been reported to become transiently exposed on the mitochondrial surface after apoptosis induction, where it is thought to promote Bax recruitment and MOMP [49, 62, 79]. Accumulation of cardiolipin potentially also facilitates the formation of Bak clusters in mitochondria [67]. Other lipids that have been frequently implicated as potent mediators of mitochondrial apoptosis are ceramides, the central intermediates of sphingolipid metabolism [39, 75]. Ceramide production was shown to increase Bax translocation to mitochondria, thereby stimulating MOMP [2, 31]. Ceramides have been demonstrated to bind VDAC2 to regulate apoptosis [18, 19]. Interaction of VDAC with ceramides was suggested to potentially block VDAC2-mediated retrotranslocation of Bax, thereby allowing its accumulation on mitochondria [19, 25, 99].

Our current understanding shows a scenario, where besides the Bcl-2 protein network, several other factors are involved in the regulation of MOMP. This includes mitochondrial dynamics, interactions of Bcl-2 family proteins with other mitochondrial proteins, membrane contact sites between mitochondria and other organelles in the cell, and potentially more, yet unknown factors. Together, these factors create an integrative signaling network that determines the functional consequence of apoptosis stimulation. Here we describe single-molecule techniques used to investigate different aspects of MOMP aiming to decipher the mechanistic secrets of how MOMP is regulated.

## Single molecule techniques to study MOMP

The mechanisms and mitochondrial alterations leading to MOMP as a key event in the execution of the intrinsic apoptotic pathway still remain a puzzle. In this regard, MOMP is a complex process with strong temporal and spatial control that proceeds out of equilibrium. Detailed insights into the structure and molecular composition of the temporal dynamics of the signaling complexes governing MOMP are necessary to understand how MOMP is mechanistically controlled. However, much of the information on how the individual molecules are involved in the execution of MOMP is masked in ensemble measurements such as pulldown assays or fluorescent microscopy techniques like fluorescence recovery after photobleaching (FRAP), fluorescence live-time imaging (FLIM) or Förster resonance energy transfer (FRET) measurements.

Luckily, the last decades have seen a rapid evolution of a number of techniques suitable to perform measurements at the single-molecule level and/or in a time-resolved manner. These include a wide range of fluorescence microscopy techniques like single-molecule imaging (SMI), localization-based super-resolution microscopy or fluorescence correlation spectroscopy. The advantage of single-molecule methods is that they allow to disentangle heterogeneities in the populations of molecules mediating MOMP, both in terms of space and time, and to connect these with function. To generate a full picture, orthogonal techniques can be applied to study protein structure and protein-protein/protein-lipid interactions like other forms of fluorescence microscopy, electron paramagnetic resonance spectroscopy, nuclear magnetic resonance spectroscopy, x-ray crystallography, electron microscopy and molecular dynamics simulations.

### Studying the interactions between Bcl-2 proteins with fluorescence correlation spectroscopy

**Fig. 2:**
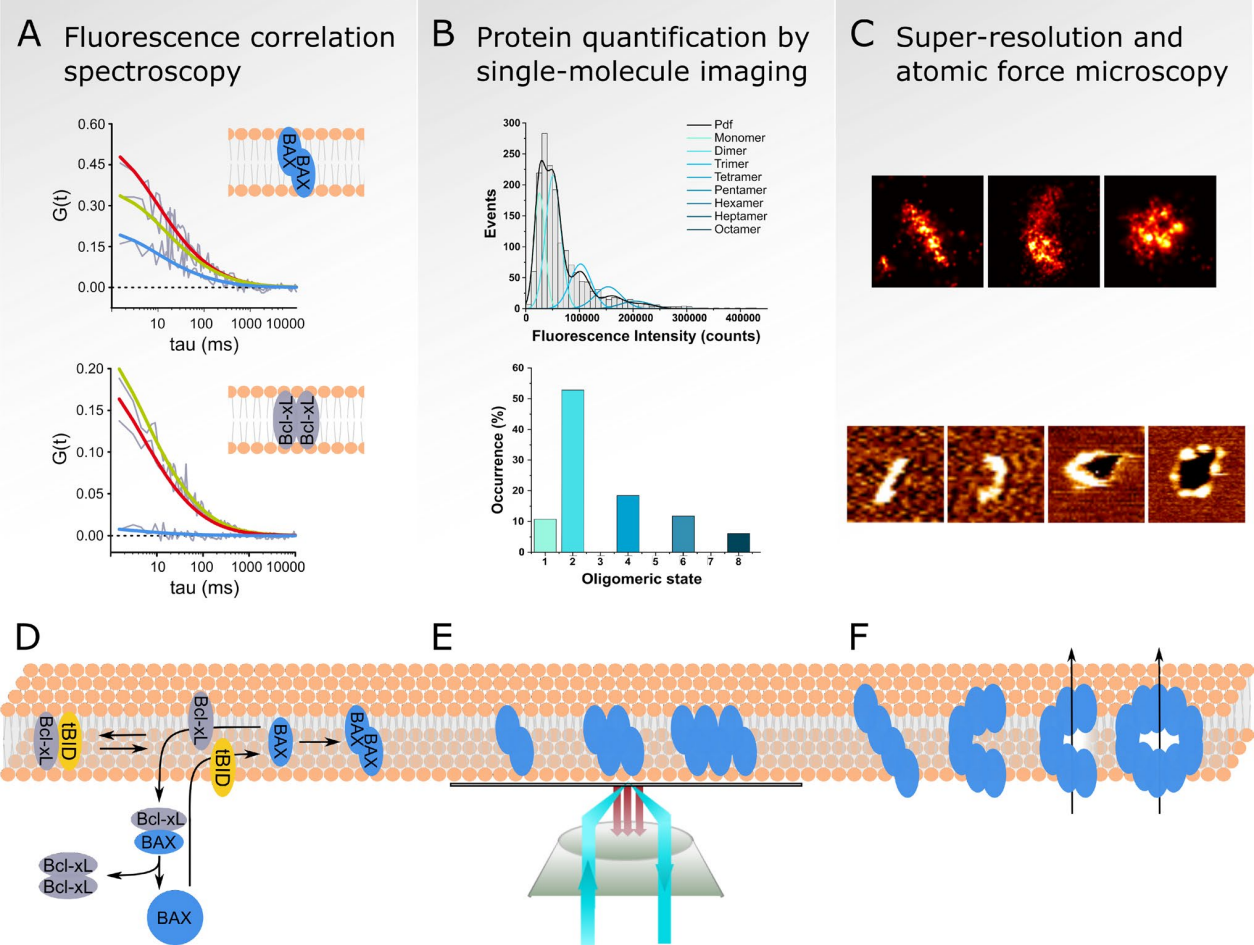
Single-molecule and nano-structural techniques to study MOMP: **a** (modified from [4]): schematic representation of fluorescence correlation spectroscopy measurements of the Bcl-2 interactome in the membrane. Representative auto-correlation (red/green) as well as cross-correlation curves (blue) are shown for Bax and Bcl-xL homodimers in the membrane as indicated. The amplitude and decay of the cross-correlation curves demonstrate interactions of Bax molecules, whereas nearly no interaction is detected for Bcl-xL homodimers. **b** Protein quantification in membranes using single-molecule imaging by TIRF microscopy in supported lipid bilayers. Representative intensity distribution histogram of fluorescent particles (top) are fitted by a probability density function (Pdf) and individual Gaussians to determine the percentage of occurrence of distinct oligomeric states within the sample (bottom). **c** Representative images of structures formed by active Bax in the membrane using dSTORM in mitochondria of apoptotic cells (top) and in supported lipid bilayers using atomic force microscopy (bottom). Membrane topography is color coded: plane membrane (orange-brown), membrane holes (black) and protein protrusions from the membrane plane (yellow). **d** Minimal Bcl-2 protein interaction model composed of Bax, Bid and Bcl-xL derived from FCS measurements. In solution, Bax is mainly monomeric, whereas Bcl-xL forms a high-affinity homo-dimeric complex. Upon activation by cBid, Bax homo-oligomerizes in the membrane additionally recruiting soluble Bax to the membrane by auto-activation (not shown). In the membrane, Bcl-xL forms complexes with cBid or Bax, with the latter one promoting retrotranslocation of Bax to the solution. **e** Schematic representation of single-molecule imaging of Bax by TIRF microscopy in supported lipid bilayers with a distribution of Bax oligomeric species based on dimer units. **f** Model of Bax structures in the membrane forming lines, arcs and rings with the ability of the arcs and rings to form a pore

The permeabilization of the MOM is a tightly controlled and highly regulated process. The overall integration of various activating (pro-apoptotic) and repressing (anti-apoptotic) stimuli in a spatially and temporally controlled manner determines the outcome of the signaling network of the Bcl-2 proteins. For this reason, it is important to understand how different proteins mechanistically orchestrate individually level to regulate MOMP. Fluorescence correlation spectroscopy (FCS) is a powerful method to quantitatively investigate the interaction of proteins. In FCS, the fluctuations of fluorescence intensity due to the diffusion of individual fluorophore-tagged proteins through the focal volume of the microscope is used to determine protein concentration and diffusion coefficients [87]. Analysis of the dynamics of the co-diffusion of proteins labelled with different fluorophores in dual-color FCS provides quantitative information on protein complex formation and binding affinities [33] (Fig. 2).

In a pioneering study of the interactions between Bcl-2 proteins at the single-molecule level, FCS was used to quantitatively compare the interaction of full-length Bid and its truncated version tBid with a *C*-terminally truncated version of Bcl-xL specifically in solution versus the membrane environment [32]. While full length Bid did not interact with Bcl-xL, the active form tBid bound to Bcl-xL in both environments. The stronger binding between both proteins in the lipid bilayer demonstrated that the membrane plays an active role by promoting complex formation between these two Bcl-2 proteins. Interestingly, peptides corresponding to the BH3 domain of Bid were shown to be able to compete for the Bid/Bcl-xL association in solution but only partially in the membrane, suggesting that the nature of the interaction is different in solution and the membrane. Based on these results, a model was proposed, where Bid and Bcl-xL interact only upon Bid cleavage after apoptosis induction promoting each other’s binding to the membrane, where they have higher affinity for each other. As a consequence, the inhibition of Bid by Bcl-xL happens mainly at the membrane, unveiling the process of membrane association as an additional regulatory stage to control MOMP. However, structures between tBID and Bcl-xL have not been determined to date, but they may reveal additional contacts between these proteins. NMR studies indicated that, in detergent, tBID exhibits an unfolded conformation with helical elements no longer interacting as observed in solution NMR structures [14, 66, 105].

Analysis of the self-assembly of Bax compared to Bcl-xL by FCS in giant unilamellar vesicles (GUVs) provided new understanding about the mechanistic differences between these two proteins, which have high sequence and structure homology but opposing apoptotic activity [5, 6]. While both Bax and Bcl-xL bound to the membrane when induced by cBid, only Bax continued to self-assemble, while Bcl-xL stayed in a low oligomeric state. Moreover, Bcl-xL and Bax exhibited unique biophysical properties in the membrane-bound state that lead to mechanistical differences in the membrane activity of both proteins. Bax creates stable long-lived pores that can be explained by an increase in membrane tension upon insertion in the membrane and oligomerization, while, at the same time, decreasing the line tension at the pore edge. Bcl-xL, on the other hand, induces transient permeability alterations of the membrane but is not able to form stable pores as it is unable to self-assemble into multimeric species and to stabilize the high curvature of the pore. Nevertheless, membrane inserted Bcl-xL inhibited Bax membrane insertion and pore activity. Correlation with MOMP using isolated mitochondria proved the physiological relevance of these findings, explaining how the different membrane activities of Bax and Bcl-xL translate to their opposing role in apoptosis.

The combination of confocal microscopy and FCS in different model membrane systems allowed to compare the membrane permeabilization and remodeling activities of Bax, cBid and Bcl-xL. Following a systematic approach, the dynamic alterations in shape and size were investigated at the single-vesicle level. For this purpose, the diffusion of large unilamellar vesicles (LUVs) estimated from the decay of the FCS auto-correlation curve allows to calculate vesicle size, whereas the amplitude of the FCS curve can be used to determine the number of vesicles present in the sample. Besides inducing membrane permeabilization, mixing cBid and Bax led to a reduction in vesicle size most likely related to membrane tethering, budding and fission [3]. This membrane remodeling effect of Bax was independent of cBid, as heat-activated Bax caused the same effects. In the absence of Bax, cBid promoted vesicle size reduction in a concentration-dependent manner but was unable to induce vesicle permeabilization. Bcl-xL could counterbalance both pore formation and vesicle fission, indicating that these processes were specifically induced by Bax and cBid. Furthermore, cBid and Bax preferentially localized to the neck region between the vesicle and the membrane budding area, in line with their potential ability to stabilize non-lamellar membrane areas of high curvature by reducing the energy required for membrane remodeling. Interestingly, such membrane structures are present as intermediate steps of both membrane fusion and fission as well as pore formation, supporting a potential regulatory role of Bax and cBid in these processes.

A quantitative analysis of a minimal Bcl-2 protein network (composed of Bax, cBid and Bcl-xL) by FCS in solution and in membranes revealed a hierarchy of protein interactions in apoptosis regulation and specifically identified membrane association as a key step in the regulatory process [4]. In solution, Bax was monomeric, whereas cBid was found as an association of its two p7 and p15 fragments or in complex with Bcl-xL. Bcl-xL, on the other hand, existed a homo-dimeric complex in solution that dissociated after addition of cBid leading to the formation of a hetero-dimeric cBid-Bcl-xL complex. FCS measurements in GUVs revealed that the interaction network of these Bcl-2 proteins strongly changed after membrane insertion. In the membrane, Bcl-xL was able to form complexes with cBid or Bax. In absence of both, Bcl-xL was present as a mixture of monomers and homo-dimeric species, indicating a lower affinity to itself than to Bax or cBid. On the other hand, Bax was present in homo-dimeric or oligomeric complexes with itself as well as in complex with cBid or Bcl-xL (Fig. 2). Once activated, Bax was able to promote its auto-activation by enhancing the recruitment of soluble Bax to the membrane and promoting oligomerization. Bax also recruited Bcl-xL to the membrane, forming a complex with it that led to the retrotranslocation of Bax to the solution, thereby inhibiting Bax oligomerization. Based on these data a new model was proposed, where the high affinity between Bcl-xL monomers in solution might be the driving force providing the necessary energy for the retro-translocation of membrane-bound Bax/Bcl-xL complexes back to solution. The fact that the affinity between Bcl-xL and cBid in the membrane was shown to be higher than the affinity between Bcl-xL and Bax, provides an additional mechanistic explanation of the pro-survival function of Bcl-xL. Formation of Bcl-xL/cBid complexes in the membrane would serve a dual purpose of keeping cBid sequestered for direct Bax activation and Bcl-xL sequestered for Bax retrotranslocation. This would prevent cBid-induced Bax activation, while it would increase the sensitivity of the system to Bax activation by other means as in a primed state.

The use of chemically controlled in vitro reconstituted systems based on model membranes has the advantage of allowing tight control of concentrations, as well as protein-to-protein and protein-to-lipid-ratios. Several experimental conditions can be tested, which provides precise analysis on the interactions and affinities between proteins. As discussed above, this strategy has been paramount for the progress in our understanding of the molecular mechanisms of Bcl-2 proteins. In living cells, however, the proteins of the Bcl-2 family not only form a fine-tuned, highly balanced interaction network, but also might be affected by yet unknown, regulatory factors. In addition, apoptosis progression is a very dynamic and irreversible process with many changes in the cellular environment that cannot be fully reproduced in vitro. For this reason, and given the limitations in the conclusions derived from in vitro experiments, the development of new single molecule methods that can be applied in the cellular context is of critical relevance. With this in mind, big efforts have been put in the implementation of FCS methods to membranes of living cells [80]. Recently, tube scanning fluorescence cross-correlation spectroscopy was introduced for the absolute quantification of diffusion and binding of fluorescently labeled proteins in the mitochondrial compartments [100]. Based on the tBid/Bcl-xL complex as a reference system, results in cells compared to in vitro studies confirming that all mitochondria-bound tBid and Bcl-xL were associated with each other, while their interaction in the cytosol was undetectable.

In summary, the unique advantage offered by FCS to quantify complex formation between proteins in an absolute manner, especially when it comes to the study of interactions specifically within the membrane environment, has been key for our understanding of the factors regulating the Bcl-2 interactome. This has far reaching consequences, for example for improving our knowledge about the mechanism of BH3-mimetics, forms of which are currently used in the clinics [20, 82, 91].

### Imaging of Bcl-2 proteins at the single molecule level

One of the major tasks in our attempt to understand the mechanism of MOMP is to shed light on the composition and structure of the apoptotic pore itself. Recent studies aimed to clarify the number of protein subunits (i.e., protein stoichiometry) that form the pore as well as the structure and size of the pore in the membrane [48, 61, 94]. Total internal reflection fluorescence (TIRF) microscopy is a powerful technique that provides high axial resolution and improved signal-to-nose-ratio compared to conventional epifluorescence microscopy. This allows imaging of fluorescently labelled proteins with single molecule sensitivity. Using TIRF microscopy in membrane model systems combined with single-particle stoichiometry analysis allowed to determine the stoichiometry of Bax oligomers at the single-molecule level [94]. In this approach, the number of molecules present in a single Bax particle is determined based on its fluorescence intensity. For this purpose, the brightness of a single fluorophore is determined by Gaussian fitting and used as a calibration to calculate the theoretical brightness of higher order oligomeric species. The distribution of fluorescence intensity measured for the individual particles in the sample is fit with a linear combination of Gaussians or probability distribution functions for each oligomeric state based on the calculated theoretical brightness and standard deviation. From this, the percentage of occurrence of each oligomeric state in the sample is determined by the area under the curve of the Gaussian fit (Fig. 2). Bax bound to cardiolipin-containing supported lipid bilayers (SLBs) when activated by incubation with the BH3-only protein cBid or after mild heating. The inability to oligomerize after membrane insertion due to unspecific interaction with the glass surface revealed that Bax initially binds to membranes as a monomer. Incubation of activated Bax in liposomes allowed the formation of oligomers prior to SLB formation and the calculation of the oligomeric state of Bax in the membrane. Surprisingly, Bax was not present as a unique oligomeric state but appeared as a broad distribution of higher-order oligomeric species based on dimer units (Fig. 2). Furthermore, it was demonstrated that the mode of activation (cBid or heat) did not affect the oligomerization behavior of Bax, whereas Bcl-xL induced the dissociation previously formed Bax oligomers. Similar results regarding the role of tBid, Bcl-xL and cardiolipin in Bax membrane insertion and oligomerization were obtained using an alternative single molecule approach based on subunit photobleaching counting, which is limited to lower oligomeric states [61]. Based on these experimental data and supported by particle-based mathematical simulation, a new model for Bax activation was proposed [94], where Bax is recruited to the membrane by cBid or by already activated, membrane-bound Bax molecules. Bax inserts into the membrane as a monomer, where it rapidly dimerizes and associates into higher-order oligomeric species based on dimer units. According to this model, active Bax in the membrane exists in a range of oligomeric states in a reversible equilibrium that can be inhibited by Bcl-xL-induced disassembly of activated Bax oligomers.

Using the same approach for quantification of protein stoichiometry, a recent study aimed to disentangle the role of oligomerization on pore formation using of two *C*-terminal mutants of Bax [Bax(T182I) and Bax(G179P)] [48]. Bax(T182I) showed much lower retrotranslocation rates from mitochondria to the cytosol measured by Fluorescent loss in photobleaching (FLIP) compared to wild-type Bax, whereas Bax(G179P) fully localized to the cytosol [48]. Although both mutants showed hampered oligomerization after activation with cBid as calculated from single molecule stoichiometry analysis, Bax(T182I) was able to mediated MOMP in apoptotic cells. However, it did not show binding nor permeabilization when it was tested in liposomes. Bax(G179P), on the other hand, was unable to execute MOMP in cells but was able to form pores in liposomes. Taken together, these findings suggested that Bax-mediated pore formation occurs independently of high-order oligomer formation and that the mitochondrial residence of Bax (determined by its retrotranslocation rate) is crucial in sensitizing the cell to apoptosis stimulation.

Analysis of protein stoichiometry in model membranes allows the tight control of sample composition and precise quantification of oligomer stoichiometry. However, it is questionable to which extent model systems faithfully recapitulate protein assembly in cells. First and foremost, the absence of additional regulatory factors present in cells as well as the artificial environment (e.g., SLBs formation on glass surfaces or Bax activation by heat) need to be taken into consideration when interpreting the results. In addition, working in the single molecule regime requires very low protein concentrations that don't cover the range of protein levels physiologically found in cells. Although mechanistically challenging, quantitative measurements of protein assembly directly in the physiological environment of the cell is necessary to complement models of protein interaction and assembly dynamics of protein complexes.

Single-molecule fluorescence is also at the core of super-resolution imaging methods like photoactivated localization microscopy (PALM) and direct stochastic optical reconstruction microscopy (dSTORM), which achieve spatial resolutions in the order of tens of nanometers and have been key to advance our understanding of MOMP. In PALM microscopy, photoactivatable or photoswitchable fluorescent proteins are used to subsequently activate the emission of individual fluorophores over several frames of acquisition [1]. This allows the detection of single fluorophores at individual time-points. 2D-Gaussian fitting of individual emitting fluorophores can be used to localize them with high precision, allowing the reconstruction of a super-resolved image. Furthermore, counting the number of detected fluorophore localizations in a region of interest provides an estimation of the number of molecules present at this specific position. Instead of photoactivatable or photoswitchable fluorescent proteins, dSTORM uses bright fluorophores with specialized photophysical properties that allow the stochastical activation and deactivation of their emission, a process commonly known as blinking [84]. Specialized excitation procedures and fluorophore-specific imaging buffers are used to fine-tune the blinking process in a way that allows the detection of individual fluorophore localizations [72].

The direct visualization of Bax pores was achieved for the first time with dSTORM [85], in parallel with a study reporting similar results visualized with another form of super-resolution microscopy called stimulated emission depletion (STED) microscopy [38]. STED is a super-resolution microscopy technique that uses a donut-shaped laser for stimulated emission depletion to create a fluorescence emitting spot smaller than the size of a diffraction limited spot [40, 41]. Visualization of GFP-Bax expressed in apoptotic HeLa cells and stained with an Alexa Fluor 647-coupled anti-GFP nanobody using dSTORM revealed a wide distribution of supramolecular structures. Besides dots and aggregates, non-random structures of Bax with a significant portion of lines, arcs and rings were identified and classified (Fig. 2). The length of lines and arcs ranged from 50 to 500 nm and the rings radius between 20 and 60 nm with an average radius of 35 nm [85]. Comparison with a non-functional mutant of Bax (Bax1-2/L-6) showed a higher proportion of undefined dot and aggregate assemblies compared to wild-type Bax and significant smaller line, arc and ring structures. In parallel, atomic force microscopy (AFM) of supported lipid bilayers containing Bax oligomers showed similar structures as Bax investigated by super-resolution microscopy including lines, arcs and rings with heterogeneous size distribution. Interestingly, the 3D topographical information provided by AFM demonstrated that almost all rings and a significant fraction of the arcs were associated with membrane pores (Fig. 2). The observation that not the whole pore rim was covered by protein density provided strong evidence for the model that Bax pores are of toroidal nature, this is, composed of both protein and lipids.

In the STED study, cluster formation of Bax at apoptotic mitochondria was analyzed in U2OS cells using fixation and immunostaining against Bax 18 h after apoptosis induction [38]. Bax assembled into large clusters at fragmented mitochondria during apoptosis with ring-like structures of diameters ranging from 200 to 800 nm. The area enclosed by Bax rings was devoid of density of Tom20 or Tom22 and associated with cytochrome *c* release, indicating that Bax rings correlate with pores or defects in the MOM. Interestingly, U2OS cells depleted for Drp1 showed significantly lower number of Bax rings and did not exhibit full cytochrome *c* release, supporting the hypothesis that Bax oligomerization and cytochrome *c* release are affected by Drp1.

Both the dSTORM and STED studies reported similar results, in that they provided the first direct visualization of Bax apoptotic pores, which, importantly, were not uniform but adopted a wide size distribution reminiscent of Bax structural flexibility. The smaller average size of the Bax rings observed in the dSTORM study compared to STED is likely linked to this flexibility and due to the different times at which the cells were fixed post-MOMP in both studies (Bax pores grow with time [81]), the different cell types used with different mitochondrial shape, as well as the fact that the spatial resolution in the STED study was worse, which induces a bias towards larger structures. Furthermore, both studies detected Bax assemblies in the form of lines, arcs and rings. The 3D analysis performed by STED in cells revealed that lines and arcs could correspond to rings visualized with a different orientation. The AFM analysis in Salvador Gallego et al., additionally showed that Bax oligomers can adopt all three shapes in the same membrane plane in vitro [85]. The most important aspect being the association of Bax arcs with membrane pores, which in the meantime has also been observed for other pore forming proteins [58, 68, 90, 93].

PALM was used to quantify Bak oligomerization *in-situ* in apoptotic cells [73]. A version of Bak genetically fused to the photoswitchable fluorescent protein mEos3 was stably expressed in mouse embryonic fibroblasts (MEFs) deficient in Bax. After UV-induced apoptosis induction, these cells were fixed and Bak clusters were analyzed using PALM microscopy with a localization precision of 19.6 nm. The average radius of Bak clusters was estimated to 111 ± 55 nm with a broad distribution between 35 and 330 nm among all detected clusters. Localization-based quantification revealed between 23 and over 2500 Bak molecules per cluster with an average of 344 ± 24 molecules, with a nearly linear increase of cluster area with the number of Bak molecules. In addition, a BaxΔGD mutant lacking two amino acids in the BH3 domain did not alter mitochondrial localization but completely abolished cluster formation and apoptotic function. A second mutant, BakΔN, lacking 20 amino acids in the N-terminal, showed higher susceptibility to apoptotic stimulation but lower number of BakΔN molecules per cluster and, on average, smaller clusters compared to wild-type Bak. These results show lack of correlation between cluster size and apoptotic activity, in agreement with the stoichiometry analysis discussed above [48]. Additionally, under all tested conditions, no pore-like structures were detected in the Bak clusters. According to the authors, this could potentially be caused by insufficient resolution due to the limited number of photons emitted from each individual fluorophore.

Because of their size in the range of 30–100 nm, the spatial resolution provided by the different super-resolution microscopy methods (based on single molecule sensitivity or not) has proven fundamental to improve our knowledge about Bax and Bak structures at apoptotic foci. Given the complex molecular architecture of these mitochondrial sites and the likely role of additional cellular factors, we are convinced that these methods have the potential to shed new light on the nanoscale organization of the apoptotic pore.

## Summary and outlook

As shown by the examples above, single molecule techniques have proven powerful tools to expand our knowledge about the regulation of MOMP and the molecular mechanisms of the Bcl-2 proteins. FCS experiments in solution and in membranes of in vitro systems as well as in organelles of living cells allows quantification of protein–protein and protein-lipid interactions, complex formation and local concentration to dissect the Bcl-2 interactome. Advances in single molecule imaging in the recent years, now allow the visualization of individual particles with improved accuracy due to increased signal-to-noise ratio and resolution. This enables to quantitatively investigate the stoichiometry of protein assemblies in reconstituted systems and in living cells in a time-resolved manner, which is key to understand MOMP execution by the apoptosis effectors Bax and Bak. Furthermore, super-resolution imaging based on single-molecule localization microscopy (such as PALM or dSTORM) provide the opportunity to investigate cellular processes with sub-diffraction resolution providing additional structural information about the molecular architecture of apoptotic foci.

To date, there are no studies published that investigate the assembly kinetics and stoichiometry of Bax and Bak in live cells during MOMP. Correlation of stoichiometry measurements with structural studies by super-resolution microscopy need to be developed to address whether the complexes formed by Bax and Bak change in properties and molecular composition before, during and after MOMP.

In the coming years, combined with complementary measurements like functional assays or methods aimed for structural determination of protein complexes (like x-ray crystallography, electron microscopy, nuclear magnetic resonance and electron paramagnetic resonance spectroscopy), single-molecule techniques promise to contribute to fully understanding the spatially and temporally confined processes involved in MOMP regulation. Given the highly dynamic, heterogeneous and irreversible nature of MOMP, correlative measurements combining several techniques to study protein interaction and assembly with functional tests and structural determination on the single-molecule will be a key development to finally answer these questions.
